# Gender Differences in Myogenic Regulation along the Vascular Tree of the Gerbil Cochlea

**DOI:** 10.1371/journal.pone.0025659

**Published:** 2011-09-29

**Authors:** Katrin Reimann, Gayathri Krishnamoorthy, Withrow Gil Wier, Philine Wangemann

**Affiliations:** 1 Anatomy & Physiology Department, Kansas State University, Manhattan, Kansas, United States of America; 2 Physiology Department, University of Maryland School of Medicine, Baltimore, Maryland, United States of America; Istituto Dermopatico dell'Immacolata, Italy

## Abstract

Regulation of cochlear blood flow is critical for hearing due to its exquisite sensitivity to ischemia and oxidative stress. Many forms of hearing loss such as sensorineural hearing loss and presbyacusis may involve or be aggravated by blood flow disorders. Animal experiments and clinical outcomes further suggest that there is a gender preference in hearing loss, with males being more susceptible. Autoregulation of cochlear blood flow has been demonstrated in some animal models *in vivo*, suggesting that similar to the brain, blood vessels supplying the cochlea have the ability to control flow within normal limits, despite variations in systemic blood pressure. Here, we investigated myogenic regulation in the cochlear blood supply of the Mongolian gerbil, a widely used animal model in hearing research. The cochlear blood supply originates at the basilar artery, followed by the anterior inferior cerebellar artery, and inside the inner ear, by the spiral modiolar artery and the radiating arterioles that supply the capillary beds of the spiral ligament and stria vascularis. Arteries from male and female gerbils were isolated and pressurized using a concentric pipette system. Diameter changes in response to increasing luminal pressures were recorded by laser scanning microscopy. Our results show that cochlear vessels from male and female gerbils exhibit myogenic regulation but with important differences. Whereas in male gerbils, both spiral modiolar arteries and radiating arterioles exhibited pressure-dependent tone, in females, only radiating arterioles had this property. Male spiral modiolar arteries responded more to L-NNA than female spiral modiolar arteries, suggesting that NO-dependent mechanisms play a bigger role in the myogenic regulation of male than female gerbil cochlear vessels.

## Introduction

**Figure 1:**
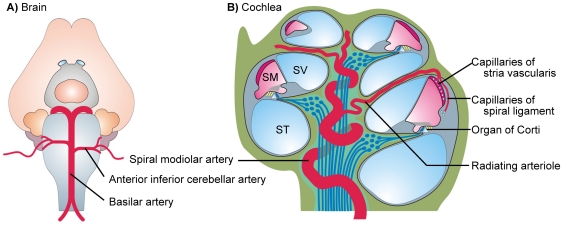
Schematic drawing of the blood vessels supplying the cochlea. Basilar artery and anterior inferior cerebellar artery ventral of the gerbil brain stem (A). Cochlea with spiral modiolar artery and radiating arteriole (B).

The cochlea receives its blood supply from the basilar artery via the anterior inferior cerebellar artery and the spiral modiolar artery [1]. The spiral modiolar artery and the radiating arterioles lie coiled up inside the modiolus and provide the capillary beds of the spiral ganglion and cochlear nerve. From there they meander along the modiolus through the interscala septa to the capillary beds in the spiral ligament and stria vascularis of the lateral wall (Fig. 1).

Blood flow regulation is critical for hearing due to the exquisite sensitivity of the cochlea to ischemia [2], [3] and oxidative stress [4], [5]. The highest density of capillaries in the cochlea is located in stria vascularis. Stria vascularis maintains the endocochlear potential which is essential for hearing [6], [7]. Many hearing disorders such as sudden sensorineural hearing loss and presbyacusis may be caused or aggravated by an impaired blood supply. Cochlear blood flow *in vivo* is significantly reduced in older gerbils [8] and gerbils suffering from age-induced hearing loss also show significant changes in the capillary beds of stria vascularis and spiral ligament [9]. All this suggests a vascular component of age-related hearing loss.

In humans, it is generally accepted that males are more susceptible to age-induced hearing loss [10], [11]. Although the conclusiveness of studies in humans is hampered by the limited control over extrinsic values such as noise and chemical/drug exposures as well as variability in disease history, animal studies in gerbils demonstrate that males are more prone to age-induced hearing loss than females [12].

Control of the vascular diameter provides the most sensitive means of blood flow control. Vascular diameter depends, in some but not all arterial vessels, on the transmural pressure [13]. Increases in the transmural pressure induce a vasoconstriction that provides a level of basal “myogenic tone”, from where a vessel could then dilate or constrict further in response to an increased or decreased demand for metabolic nutrients from the perfused tissue. Thus, myogenic regulation of blood vessels ensures constant tissue perfusion despite variations of systemic blood pressure and thereby contributes to autoregulation of blood flow *in vivo*.

*In vivo* measurements of cochlear blood flow revealed that flow is largely maintained in the face of variant blood pressures [14], [15] although, the autoregulatory response of cochlear blood flow was weaker compared to cortical flow [16]. The question, where do autoregulatory mechanisms exist along the vascular tree supplying the cochlea, remained undetermined.

The aim of the present study was to determine the site of myogenic regulation along the vascular tree of the cochlea, beginning with the basilar artery, followed by the anterior inferior cerebellar artery, the spiral modiolar artery and radiating arterioles that feed the capillary beds in the modiolus and the lateral wall and to determine gender-dependent differences in regulation.

## Materials and Methods

### Animals

All procedures involving animals were approved by the Institutional Animal Care and Use Committee at Kansas State University (IACUC#: 2613 and 2961). Male and female gerbils and females rats (Charles River, Wilmington, MA) were anesthetized with tribromoethanol (560 mg/kg i.p.) and decapitated prior to harvest of vascular tissues. The spiral modiolar artery and radiating arterioles were isolated from the cochlea, the basilar artery and the anterior inferior cerebellar arteries were isolated from the ventral surface of the brain stem [17], [18] and a branch of the mid-cerebral artery was isolated from the dorsal surface of the cerebral cortex. Arterial segments were transferred into a custom-built bath chamber holding a volume of 120 µl that was mounted on the stage of an inverted microscope (Axiovert 200, Carl Zeiss, Göttingen, Germany).

### Solutions

The dissecting solution and the control solution contained (in mM) 150 NaCl, 5 HEPES, 3.6 KCl, 1 MgCl_2_, 1 CaCl_2_ and 5 glucose, pH 7.4. In some experiments, L-N^G^-Nitro-Arginine (L-NNA, Sigma, St. Louis, USA) was added to this solution. Ca^2+^-free solution contained (in mM) 150 NaCl, 5 HEPES, 3.6 KCl, 1 MgCl_2_, 1 EGTA and 5 glucose, pH 7.4.

### Perfusion

**Figure 2:**
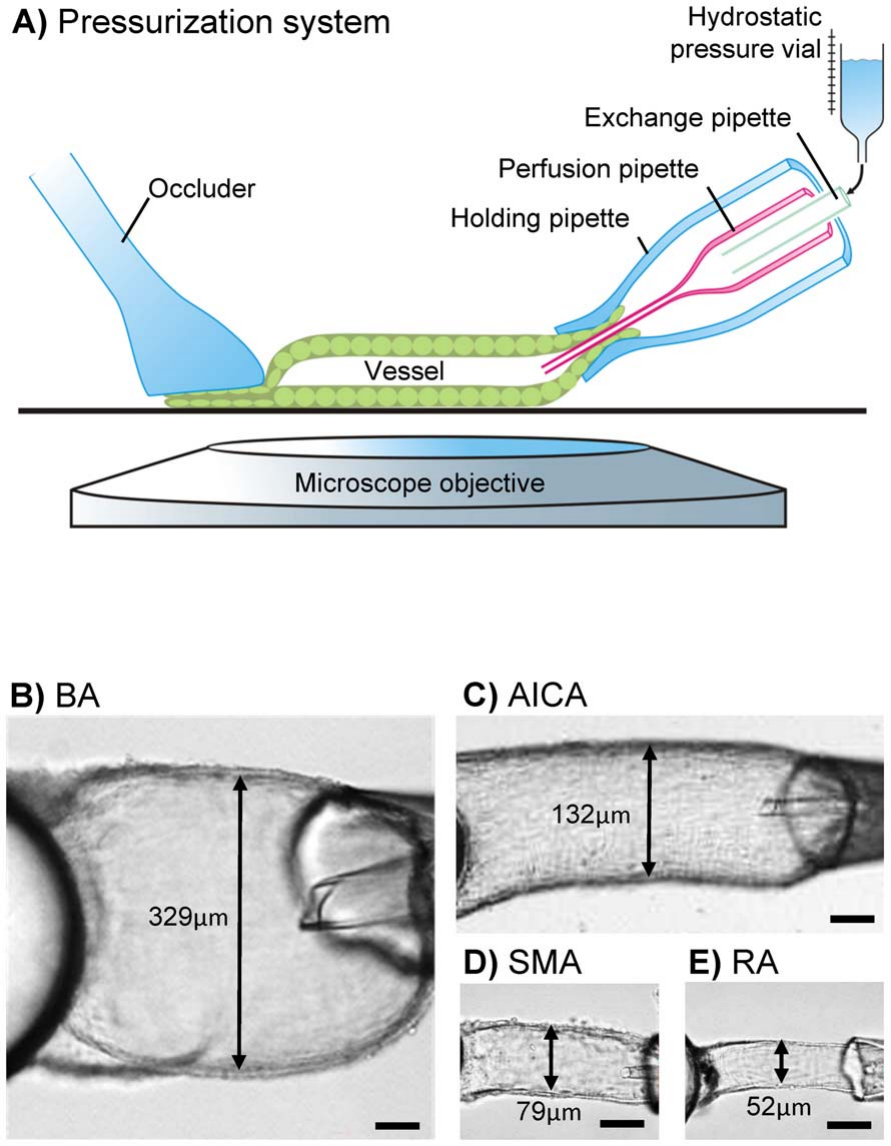
Pressurization of vessels using the concentric pipette system. Schematic drawing of pressurization system (A) showing the vessel at the right end linked to a concentric pipette system with a hydrostatic pressure column of variable height to control transmural pressure while the left end of the vessel is occluded by pressing the vessel against the bottom of the bath chamber with a plan glass holder. Pressurized basilar artery (B), anterior inferior cerebellar artery (C), spiral modiolar artery (D), and radiating arteriole (E), scale bars = 50 µm.

Arteries were pressurized using a motorized system of concentric pipettes (Wangemann Instruments, Kansas State University). The pipette system linked the vessel at one end to a hydrostatic pressure column of variable height while the other end of the vessel was occluded by pressing the vessel against the bottom of the bath chamber with a plan glass holder (Fig. 2). Experimental procedures were similar to those described earlier [17]. Pipettes were prepared on a custom-built micro-forge. Pressurized arteries were superfused at a rate of 1 ml min^−1^. A temperature of 37°C was maintained by a triple heating system, controlling the temperature of the bath chamber, the microscope objective and the superfusate.

### Measurement of vascular diameter

Pressurized vessels were imaged by laser scanning microscopy (LSM 510 Meta, Carl Zeiss) and the inner diameter (ID) was detected using a custom written data acquisition program (W. Gil Wier, University of Maryland). Data were analyzed using a custom written analysis program (P. Wangemann, Kansas State University). Percent myogenic tone was calculated as (ID_passive_–ID_active_) × 100/ID_passive_, with ID_passive_ detected in Ca^2+^-free solution and ID_active_ detected in control solution containing 10 µM L-NNA.

### Estimation of the pressure dependence of flow

The pressure dependence of flow was estimated based on the law of Hagen-Poiseuille (Equation 1) which links flow rate [mm^3^/s] in vessels to the vessel radius *r* [mm], the intramural pressure difference *ΔP* [Pa] over a given length *l* [mm] of the vessel, and to the viscosity *η* [Pa.s] of the luminal fluid. With the goal to compare data from different vessels, we substituted the transmural pressure for the intramural pressure and set the length to 1 mm.
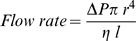
(Equation 1)


### Statistics

The number of experiments (n) represents the number of vessel segments. Data are presented as average ± SEM. Significant differences in tone between male and female arteries were obtained by performing 2-way repeated measures ANOVA and significance was determined at p<0.05. Data were normalized for presentation.

## Results

### Validation of experimental procedures

**Figure 3:**
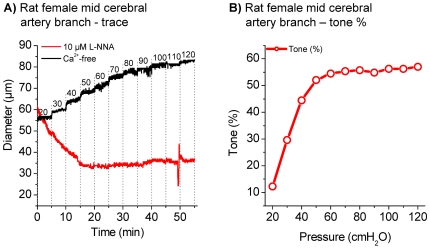
Diameter and myogenic tone in female rat mid-cerebral artery territory branch. Original trace of female rat mid-cerebral artery territory branch in 10 µM L-NNA and Ca^2+^-free solution (A). Calculated myogenic tone in %, n = 1 (B).

Blood vessels of different origin vary in their ability to generate vascular tone. *A priori*, the observation of a small or absent vascular tone in a vessel that has not been studied before can be an intrinsic property of that vessel or can be the result of unfavorable experimental procedures. As validation of our experimental procedures, we demonstrated vascular tone development in a small arteriole from the mid-cerebral artery territory of female rat which is known to develop a robust tone of ∼40 and ∼50% in the presence of nitric oxide synthase inhibitors [19], [20]. We pressurized the arteriole (∼80 µm inner diameter) from the mid-cerebral artery territory and superfused the vessel with control solution to which the nitric oxide synthase inhibitor L-NNA (10 µM) had been added. With increasing transmural pressures the arteriole constricted vigorously (Fig. 3). The passive diameter was determined by superfusion with Ca^2+^ free solution and myogenic tone was calculated, see Methods. At a transmural pressure of 60-120 cmH_2_O, a myogenic tone of ∼55% was observed (Fig. 3). This value is similar in magnitude to the myogenic tone of ∼52% that was reported previously [20], which validates our experimental procedures. All further experiments were performed on vessel segments isolated from gerbils.

### Passive diameters at reference pressure

The mean arterial blood pressures in the gerbil basilar artery, anterior inferior cerebellar artery, spiral modiolar artery and radiating arteriole were estimated to be 62, 58, 56 and 56 cmH_2_O, respectively, based on the gerbil mean arterial blood pressure of ∼102 cmH_2_O [21], [22] and on pressure measurements in the vascular tree of cat pial arteries [23]. Based on these estimations, a pressure of 60 cmH_2_O was chosen as reference pressure for all gerbil vessels. At this reference pressure, the passive inner diameter of pressurized vessels was determined by superfusion with Ca^2+^ free solution and found to be 314 µm±8 (n = 9) for the basilar artery, 131 µm±5 (n = 13) for the anterior inferior cerebellar artery, 78 µm±3 (n = 13) for the spiral modiolar artery and 51±2 (n = 8) for radiating arterioles. No gender differences were apparent in these measurements. Next, pressurized arteries were subjected to pressure-step protocols.

### Pressure dependence of vascular diameter and tone

In response to increasing transmural pressures, the spiral modiolar artery and radiating arterioles developed very little tone when perfused with control solution in the absence of L-NNA (*data not shown*). We hypothesized that myogenic tone development may be masked by high endogenous levels of nitric oxide given that diameter measurements were made *in vitro* in the absence of red blood cells that scavenge nitric oxide [24], [25]. Moreover, male and female animals differ in the levels of endothelial NO production [26]. To observe gender differences, comparable conditions can be achieved by blocking eNOS [19], [27], [28]. Hence the active diameter in all further experiments was measured in control solution with10 µM L-NNA, which is a potent NOS inhibitor. The passive diameter was determined in Ca^2+^ free solution and the myogenic tone was calculated as described in Methods.

**Figure 4:**
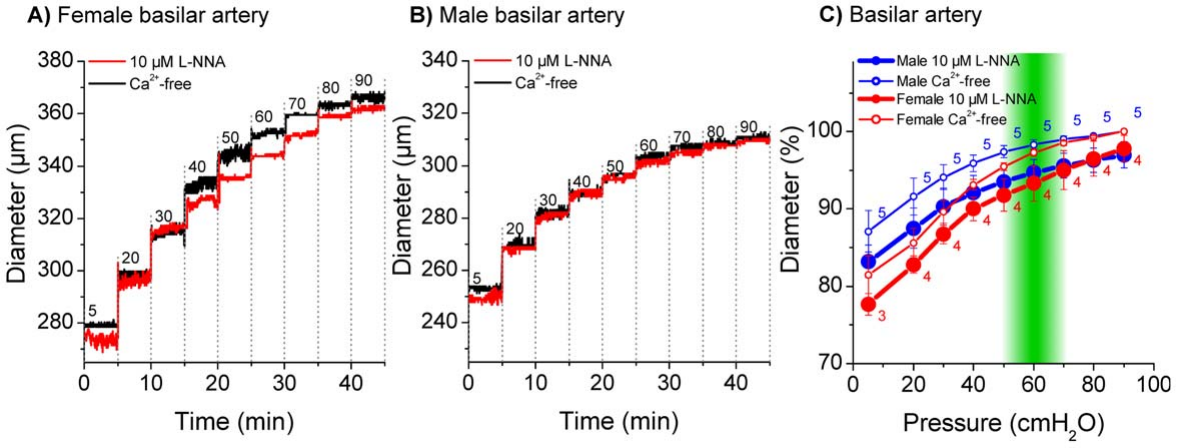
Pressure dependent diameter of female and male basilar artery. Original traces of female (A) and male (B) basilar artery in 10 µM L-NNA and Ca^2+^-free solution. Diameter in % in 10 µM L-NNA and Ca^2+^-free solution (C), % values were normalized to maximal diameter measured in Ca^2+^-free at 90 cmH_2_O. 100% = 329.2 µm±17.5 in Ca^2+^-free at 90 cmH_2_O in female animals and 313.8 µm±7.7 in Ca^2+^-free at 90 cmH_2_O in male animals.

**Figure 5:**
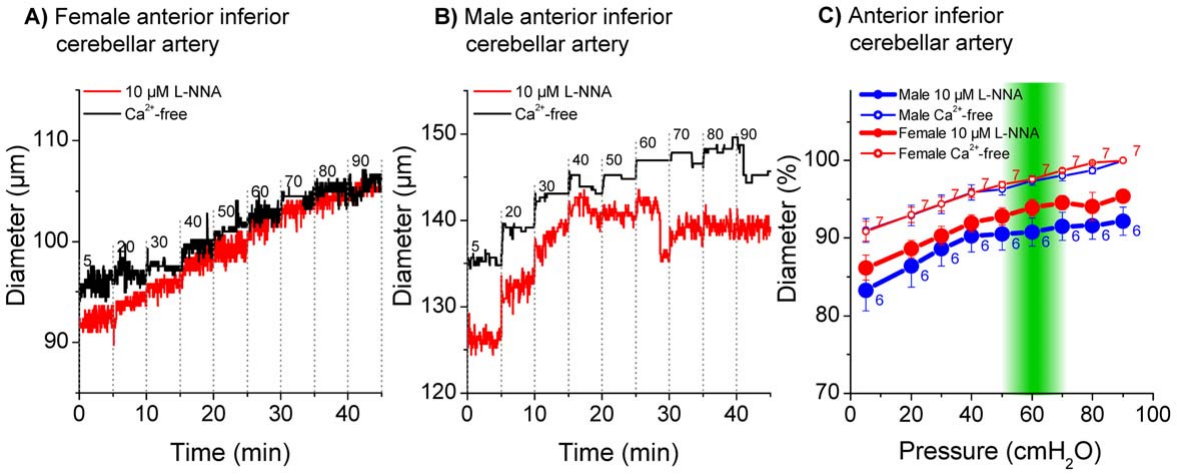
Pressure dependent diameter of female and male anterior inferior cerebellar artery. Original trace of female (A) and male (B) anterior inferior cerebellar artery in 10 µM L-NNA and Ca^2+^-free solution. Diameter in % in 10 µM L-NNA and Ca^2+^-free solution (C), % values were normalized to maximal diameter measured in Ca^2+^-free at 90 cmH_2_O. 100% = 134.6 µm±6 in Ca^2+^-free at 90 cmH_2_O in female animals and 132.8 µm±7.6 in Ca^2+^-free at 90 cmH_2_O in male animals.

**Figure 6:**
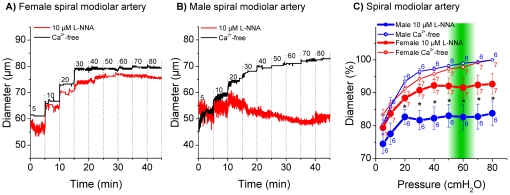
Pressure dependent diameter of female and male spiral modiolar artery. Original trace of female (A) and male (B) spiral modiolar artery in 10 µM L-NNA and Ca^2+^-free solution. Diameter in % in 10 µM L-NNA and Ca^2+^-free solution (C), % values were normalized to maximal diameter measured in Ca^2+^-free at 80 cmH_2_O. 100% = 81.2 µm±4.5 in Ca^2+^-free at 90 cmH_2_O in female animals and 77.7 µm±2.9 in Ca^2+^-free at 90 cmH_2_O in male animals. “*” indicates statistical significance (p<0.05) by ANOVA (2-factor repeated-measures).

**Figure 7:**
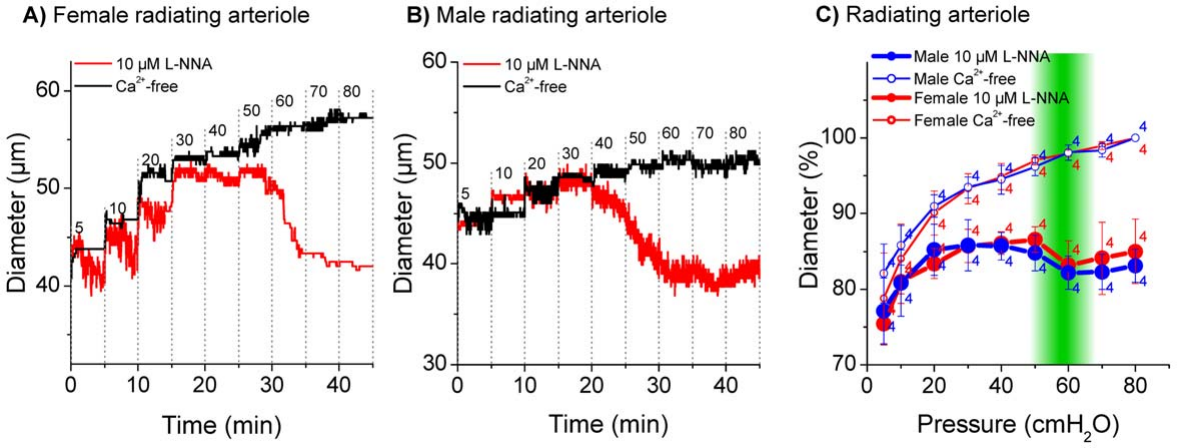
Pressure dependent diameter of female and male radiating arteriole. Original trace of female (A) and male (B) radiating arteriole in 10 µM L-NNA and Ca^2+^-free solution. Diameter in % in 10 µM L-NNA and Ca^2+^-free solution (C), % values were normalized to maximal diameter measured in Ca^2+^-free at 80 cmH_2_O. 100% = 52.4 µm±3.3 in Ca^2+^-free at 90 cmH_2_O in female animals and 53.2 µm±1.7 in Ca^2+^-free at 90 cmH_2_O in male animals.

**Figure 8:**
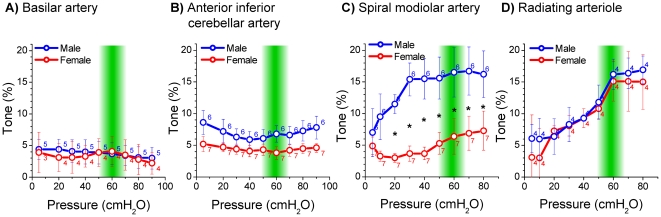
Myogenic tone development along the cochlear vascular tree in male and female gerbil. The myogenic tone at the reference pressure of 60 cmH_2_O was 4±2% in male (n = 5) and 4±1% in female (n = 4) basilar arteries (A), 7±1% in male (n = 6) and 4±1% in female (n = 7) anterior inferior cerebellar arteries (B), 17±4% in male (n = 6) and 6±3% in female (n = 7) spiral modiolar arteries (C), and 16±2% in male (n = 4) and 15±3% in female (n = 4) radiating arterioles (D). “*” indicates statistical significance (p<0.05) by ANOVA (2-factor repeated-measures).

Measurements in the basilar artery and in anterior inferior cerebellar arteries from male and female gerbils revealed only small differences between active and passive diameters (Figs. 4 and 5). On the other hand, measurements in the spiral modiolar artery demonstrated significant differences between male and female gerbils. The active diameter in male spiral modiolar arteries was significantly lesser than in female spiral modiolar arteries at pressures 20 to 80 cmH_2_O (Fig. 6). In contrast, the active diameters of male and female radiating arterioles were similar (Fig. 7). The calculated myogenic tone at the reference pressure of 60 cmH_2_O was 4±2% in male and 4±1% in female basilar arteries, 7±1% in male and 4±1% in female anterior inferior cerebellar arteries, 17±4% in male and 6±3% in female spiral modiolar arteries, 16±2% in male and 15±3% in female radiating arterioles (Fig. 8).

## Discussion

We evaluated myogenic tone in arterial vessel segments from the vascular tree of the gerbil cochlea that ranged in inner diameter from ∼310 to ∼50 µm. Measurements in vessels from male and female gerbils revealed that vessels from both genders develop ∼15% myogenic tone in radiating arterioles, which are pre-capillary arterioles with an inner diameter of ∼50 µm. Significant gender differences, with 17% tone in male and 6% tone in female vessels, were observed in the spiral modiolar artery, which is an arteriole with an inner diameter of ∼80 µm that is located immediately upstream of the radiating arterioles. Arteries located higher upstream, including the anterior inferior cerebellar artery with an inner diameter of ∼130 µm and the basilar artery with an inner diameter of ∼310 µm, developed a gender-independent tone of 4 to 7%.

The myogenic tone reported in the present study was recorded in the presence of the nitric oxide synthase inhibitor L-NNA, which is an established procedure to uncover myogenic tone in isolated vessel preparations [19], [27], [28], [29]. Nitric oxide levels in isolated vessel segments are likely to be different than *in vivo*. On the one hand red blood cells provide high concentrations of the scavenger hemoglobin that dissipates the nitric oxide generated by the endothelial cells [24], [25]. On the other hand shear stress can induce nitric oxide production by endothelial cells [30]. In isolated pressurized arteries, under no-flow conditions, both red blood cell scavenging and shear stress mediated NO production are absent. Evidence that nitric oxide controls cochlear blood flow comes from the observation that the nitric oxide synthase inhibitor L-NAME decreased cochlear blood flow *in vivo* [31] and evidence that nitric oxide levels *in vivo* are below levels that produce a saturated vasodilation comes from the observations that the nitric oxide donor sodium nitroprusside increased cochlear blood flow *in vivo* [32]. Consistent with the findings of the present study, *in vivo* application of the nitric oxide donor eliminated autoregulation of cochlear blood flow in the guinea pig [14]. The observation that the nitric oxide synthase inhibitor uncovered a significantly larger myogenic tone in spiral modiolar arteries from male compared to female gerbils suggest that NO-dependent mechanisms are more potent in males than in females. This finding is in contrast to observations in cerebral vessels where nitric oxide synthase inhibitors uncovered a significantly larger myogenic tone in females than in male rats [19].

Myogenic regulation of tone is a prominent mechanism by which arterioles change diameter to keep blood flow steady in the face of fluctuating blood pressures. It is one of the mechanisms that contribute to the autoregulation of blood flow. Autoregulation has been demonstrated in *in vivo* measurements of cochlear blood flow [14], [15], [33], [34]. The observation that radiating arterioles gender-independently developed the largest myogenic tone in the cochlear vascular tree suggests that radiating arterioles are the main site of myogenic regulation of cochlear blood flow.

**Figure 9:**
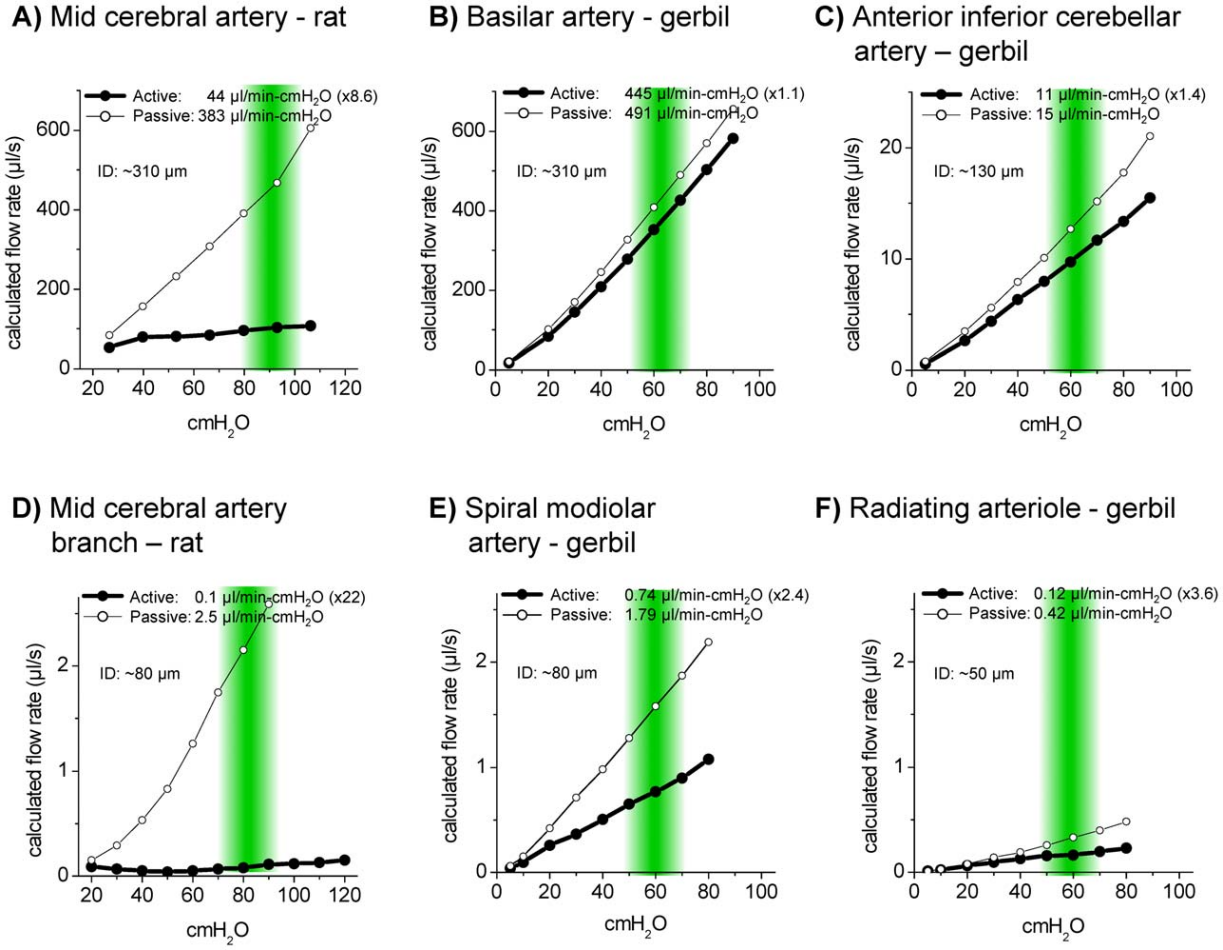
Calculated flow rate for brain arteries from rat and gerbil and cochlea arteries from gerbil. Male rat mid-cerebral artery (A) taken from Geary et al 1998 [19], male gerbil basilar artery (B) male gerbil anterior inferior cerebellar artery (C), female rat mid-cerebral artery territory branch (D), male gerbil spiral modiolar artery (E), male gerbil radiating arteriole (F). Green bars represent estimated physiological pressure range for each vessel according to which active and passive slope were calculated. Slope is given in µl/min –cmH_2_O. For active slope diameter was obtained in 10 µM L-NNA, for passive slope diameter was obtained in Ca^2+^-free solution.

We estimated the pressure-dependence of flow by transforming measured diameter values into calculated flow rates using the Hagen-Poiseuille equation (Fig. 9). Flow estimations were made based on data obtained in the present study and, for comparison, based on data obtained from mid-cerebral arteries of male rats [19]. As expected, calculated flow under active and passive conditions was linearly related to pressure. The slope of the calculated flow/pressure relationship was larger in passive vessels than in active vessels. The slope of the calculated flow/pressure relationship in active arterioles obtained from the mid-cerebral territory that supply the cerebral cortex approached zero, similar to the slope of autoregulatory curves obtained from blood flow measurements *in vivo*. Similarly, the slope of the calculated flow/pressure relationship of active radiating arterioles was close to zero suggesting that flow in this cochlear vessel is regulated by myogenic mechanisms. A comparison of the slope under active and passive conditions provides a measure for the magnitude by which blood flow can be increased on demand. In radiating arterioles and in the spiral modiolar artery, passive slopes were 3.6 and 2.4-fold larger than active slopes, respectively. A similar 3-fold difference in blood flow rates between passive and active conditions has been reported based on *in vivo* measurements of cochlear blood flow [14]. In contrast, passive slopes for arterioles in the mid-cerebral territory and the upstream mid-cerebral artery were 32 and 9-fold larger than active slopes, respectively. The estimated lower capacity of the cochlear vessels to increase blood flow is consistent with a merely 2-fold increase in glucose uptake that was observed in the presence of loud noise compared to silence [35]. Although cochlear function is dependent on glucose and highly sensitive to ischemia, there is little difference in the metabolic need between noise and silence [2], [36], [37].

An interesting feature of cochlear vessels is their remarkable length compared to the organ size. The spiral modiolar artery and the radiating arterioles lie coiled up inside the modiolus and provide the capillary beds of the spiral ganglion and cochlear nerve. The radiating arterioles then meander along the modiolus through the interscala septa to the capillary beds in the spiral ligament and stria vascularis of the lateral wall (Fig. 1). The vascular tree of cochlea measures ∼11 mm including ∼7 mm of spiral modiolar artery and ∼3 mm of radiating arterioles in the gerbil [18]. The estimated distance from a segment of the mid-cerebral artery until it branches into its capillary bed is 2–3 mm. It appears that the remarkable length of the cochlear vessels compensates for the limited myogenic tone of ∼15% to achieve a substantial reduction in blood flow. Indeed, vessels in the territory of the mid-cerebral artery that range in inner diameter from 50 to 300 µm develop under similar conditions myogenic tones of 40–50% (Fig. 3) [19], [38]. The considerably larger myogenic tone provides cerebral vessels with an apparently larger capacity to increase blood flow on demand.

The observation of significant gender differences in blood flow regulation in the spiral modiolar artery parallels the gender differences in age-related hearing loss that has been established in the gerbil model, although the observed parallelism does not provide evidence for causality [12]. The estimation that cochlear blood vessels have a limited (2.4–3.6-fold) capacity to increase blood flow is consistent with the dynamic range of metabolic demand between noise and silence [35]. However, the limited capacity to increase the delivery of metabolic substrates contrasts with the >9-fold dynamic range of stria vascularis to increase rates of ion transport [39]. It is therefore conceivable that conditions may arise where the metabolic demand of stria vascularis exceeds the capacity of the vasculature to deliver. Such mismatches between supply and demand would lead to ischemic events. Ischemic events can be expected to cause oxidative stress in the stria vascularis, a key pathophysiological mechanism leading to loss of KCNJ10 channels, which generate the endocochlear potential essential for normal hearing. [4], [40].
